# The effect of ectomycorrhizal fungal exposure on nursery-raised *Pinus sylvestris* seedlings: plant transpiration under short-term drought, root morphology and plant biomass

**DOI:** 10.1093/treephys/tpae029

**Published:** 2024-03-12

**Authors:** Gonzalo De Quesada, Jie Xu, Yann Salmon, Anna Lintunen, Sylvain Poque, Kristiina Himanen, Jussi Heinonsalo

**Affiliations:** Department of Forest Sciences, Institute for Atmospheric and Earth System Research (INAR), Faculty of Agriculture and Forestry, University of Helsinki, Latokartanonkaari 7, PO Box 27, FI-00014 Helsinki, Finland; Department of Forest Sciences, Institute for Atmospheric and Earth System Research (INAR), Faculty of Agriculture and Forestry, University of Helsinki, Latokartanonkaari 7, PO Box 27, FI-00014 Helsinki, Finland; Department of Forest Sciences, Institute for Atmospheric and Earth System Research (INAR), Faculty of Agriculture and Forestry, University of Helsinki, Latokartanonkaari 7, PO Box 27, FI-00014 Helsinki, Finland; Institute for Atmospheric and Earth System Research (INAR), Faculty of Sciences, University of Helsinki, Gustaf Hällströmin katu 2, PO Box 64, FI-00014 Helsinki, Finland; Department of Forest Sciences, Institute for Atmospheric and Earth System Research (INAR), Faculty of Agriculture and Forestry, University of Helsinki, Latokartanonkaari 7, PO Box 27, FI-00014 Helsinki, Finland; Institute for Atmospheric and Earth System Research (INAR), Faculty of Sciences, University of Helsinki, Gustaf Hällströmin katu 2, PO Box 64, FI-00014 Helsinki, Finland; National Plant Phenotyping Infrastructure, HiLIFE, Faculty of Agriculture and Forestry, University of Helsinki, Latokartanonkaari 7, 00790 Helsinki, Finland; Department of Agricultural Sciences, Viikki Plant Science Centre, University of Helsinki, Latokartanonkaari 7, 00790 Helsinki, Finland; National Plant Phenotyping Infrastructure, HiLIFE, Faculty of Agriculture and Forestry, University of Helsinki, Latokartanonkaari 7, 00790 Helsinki, Finland; Organismal and Evolutionary Biology Research Programme, Faculty of Biological and Environmental Sciences, Biocenter Finland, University of Helsinki, Viikinkaari 1, 00790 Helsinki, Finland; Department of Forest Sciences, Institute for Atmospheric and Earth System Research (INAR), Faculty of Agriculture and Forestry, University of Helsinki, Latokartanonkaari 7, PO Box 27, FI-00014 Helsinki, Finland

**Keywords:** EMF functional traits, exploration type, hydrophobicity, specific root length, stress physiology

## Abstract

Drought is a major environmental stressor that limits seedling growth. Several studies have found that some ectomycorrhizal fungi may increase the drought tolerance of nursery-raised seedlings. However, the precise role that different ectomycorrhizal fungi species play in drought tolerance remains unclear. We evaluated the transpiration rate of *Pinus sylvestris* seedlings under drought stress in greenhouse conditions by exposing seedlings to 10 ectomycorrhizal fungi species, with different functional traits (exploration type and hydrophobicity), and to 3 natural soil inoculums. We measured the transpiration and water potential of the seedlings during a 10-day drought period and a 14-day recovery period. We then analyzed their root morphology, stem, needle, root biomass and needle chlorophyll fluorescence. We showed that exposing seedlings to ectomycorrhizal fungi or soil inoculum had a positive effect on their transpiration rate during the driest period and through the recovery phase, leading to 2- to 3-fold higher transpiration rates compared with the nonexposed control seedlings. Seedlings exposed to medium-distance ectomycorrhizal fungi performed better than other exploration types under drought conditions, but ectomycorrhizal fungi hydrophobicity did not seem to affect the seedlings response to drought. No significant differences were observed in biomass accumulation and root morphology between the seedlings exposed to different ectomycorrhizal fungi species and the control. Our results highlight the positive and species-specific effect of ectomycorrhizal fungi exposure on drought tolerance in nursery-raised Scots pine seedlings. The studied ectomycorrhizal fungi functional traits may not be sufficient to predict the seedling response to drought stress, thus physiological studies across multiple species are needed to draw the correct conclusion. Our findings have potential practical implications for enhancing seedling drought tolerance in nursery plant production.

## Introduction

Ectomycorrhizal fungi (EMF) are important belowground components in boreal ecosystems. Most of the nitrogen in boreal forest soils cannot be directly utilized by plants due to being bound in organic form and only being available through microbial decomposition (Toljander et al. 2006, Manzoni et al. 2010). Ectomycorrhizal fungi play an important role in nutrient mobilization and transport from organic matter to host trees (Read 1991, Chalot and Brun 1998, Bödeker et al. 2014, Heinonsalo et al. 2015, Op De Beeck et al. 2018). Thus, plant roots form symbiotic EMF associations to enhance nutrient uptake in boreal forests (Van Hees et al. 2006, Baskaran et al. 2017). Ectomycorrhizal fungi, and their interaction with the soil microbial community, are also reported to increase plant tolerance to biotic and abiotic stresses, such as drought (Chakravarty and Unestam 1987, Sánchez et al. 2001, Luo et al. 2011; Aryal et al. 2020, Allsup et al. 2023). In boreal ecosystems, soil communities are heterogeneous, and their diversity is tightly associated with soil fertility gradients (Sterkenburg et al. 2015), and forest management can bring significant changes to the structure of soil microbial communities (Wu et al. 2019, Zhou et al. 2020).

Boreal forests face new challenges caused by changes in water availability due to climate change, and the occurrence and intensity of summer droughts in particular are predicted to increase in many regions (Venäläinen et al. 2020). Drought is a major environmental stressor, which leads to decreased plant production, an increased risk of biotic damages, and ultimately to plant dieback (Carnicer et al. 2011, Oliva et al. 2014, Caminero et al. 2018). The reduction of stomatal conductance is one of the first effects of drought on plant growth. This is an adaptation mechanism for reducing water loss through transpiration, but in turn it restrains the carbon assimilation process by limiting carbon uptake from the atmosphere (Irvine et al. 1998). Thus, transpiration rate is widely used to assess drought tolerance in plants, as it correlates with stomatal conductance, assimilation and water-use efficiency. Tree seedlings are particularly sensitive to drought stress due to their developing root systems. Drought events impair seedling growth, leaving them at a disadvantage in highly competitive environments such as closed-canopy forests (Castro et al. 2004, Shovon et al. 2021). This has the potential to affect reforestation and afforestation efforts that rely on nursery-raised seedlings, and consequently impact forest tree regeneration. The survivability of seedlings in field conditions is highly dependent on environmental factors as well as their morphological and physiological attributes, such as shoot height, stem diameter, root growth and stress tolerance (Grossnickle and MacDonald 2018). However, high seedling mortality rates associated with extensive drought periods are reported (Helenius et al. 2002, Thorpe and Timmer 2005). Tree seedling exposure to EMF in nursery conditions has been proven to increase growth (Vaario et al. 2009, Sanchez-Zabala et al. 2013, Sebastiana et al. 2013), but few studies exist on the effect of EMF exposure on seedlings water relations under drought conditions (Lehto and Zwiazek 2011).

The effects of EMF on plant water relations have been a controversial topic. While several studies have found that EMF increase plant drought tolerance in seedlings (Alvarez et al. 2009, Beniwal et al. 2010, Yin et al. 2018), other studies observed no significant differences on water relations under drought conditions between inoculated and non-inoculated seedlings (Lehto 1992, Sebastiana et al. 2018). Additionally, studies comparing multiple EMF species are uncommon, and the few studies we are aware of report contrasting results on the effects of EMF under drought conditions (Parke et al. 1983, Boyle and Hellenbrand 1991, Kipfer et al. 2012). The effects of EMF on plant drought tolerance are potentially attributed to their ability to increase plant root extension and increase root system surface area (Lehto and Zwiazek 2011). However, the mechanisms responsible for the possible increase in plant water uptake are not fully understood, but some studies suggest that this could be attributed to their hyphal water uptake ability (Duan et al. 1996) and to the production and accumulation of sugar alcohols, which protect the EMF structure in dry conditions (Shi et al. 2002).

The hyphal water uptake ability of various EMF species is potentially linked to their exploration type, a morphological attribute based on mycelium development and structure closely related to their ability to reach different distances from the host root tips (Agerer 2001). Based on the exploration distance from the root tips, four exploration types of EMF were defined by Agerer (2001): contact, and short, medium and long distance. Ectomycorrhizal fungi species with longer exploration-type and a hydrophobic mantle are assumed to be more efficient at water transport, potentially benefiting plants under drought conditions (Castaño et al. 2023). These fungi are considered to possess a better foraging capacity for nutrients and water through hyphae that aggregate into long-reaching rhizomorphs (Garcia de Jalon et al. 2020). However, recent studies question the use of exploration type as soil foraging predictors (Jörgensen et al. 2023). Furthermore, the EMF hydrophobicity, classified by Unestam (1991) into hydrophilic and hydrophobic, is pivotal in water transport within the extraradical mycelium (Unestam and Sun 1995). Hydrophilic EMF can transport water in the apoplast, suggesting higher efficiency (Prieto et al. 2016), whereas hydrophobic EMF form complex mycelial cords that transport water in the symplast (Lehto and Zwiazek 2011). However, hydrophobic EMF are not entirely water-repellent; they form a water-repellent wall mitigating water loss in arid conditions, while featuring hydrophilic tips enabling the uptake of soluble nutrients from the soil (Unestam and Sun 1995). Moreover, typical drought-adapted EMF species have hydrophobic rhizomorphs to prevent water loss (Agerer 2001), as observed in *Suillus* spp. and *Rhizopogon* spp., which are considered drought indicator species (Castaño et al. 2023). Although numerous species have yet to be thoroughly studied, the exploration type and hydrophobicity of EMF could potentially affect the plant’s morphological and physiological response to drought conditions.

Plant responses to drought, both morphological and physiological, are critical indicators of growth and survival (Smithwick et al. 2013). Photosynthesis is an important physiological process responsible for carbohydrate synthesis that is notably impacted by drought, particularly quantum yield and non-photosynthetic quenching (Lu and Zhang 1999, Junker et al. 2017, Nosalewicz et al. 2022). Maximum quantum yield (F_v_/F_m_) represents the efficiency of Photosystem II in converting light into sugars (Emerson 1958, Evans 1987), and non-photochemical quenching (NPQ) regulates photosynthesis under high light intensity (Horton et al. 1994, Muller et al. 2001). Similarly, root morphology traits, such as specific root length (SRL), are important for plant growth and survival (Jinfei et al. 2020) and are also sensitive to abiotic stress. Accordingly, pine seedlings exposed to prolonged drought have been reported to show higher belowground biomass relative to aboveground biomass than non-stressed plants (Aaltonen et al. 2016), and, in a meta-data analysis, Zhou et al. (2018) found that drought significantly reduces root length and root length density. Furthermore, biotic factors like EMF symbiosis impact root morphology, e.g. Sun et al. (2010) found that EMF increases root diameter and decreases root length and SRL in larch trees under two nitrogen fertilization treatments. The effects of EMF on plant physiology can vary depending on EMF species (Gehring et al. 2017). Additionally, previous studies have shown that soil inoculation from different ecosystems can lead to changes in the soil microbial community and plant development (Wubs et al. 2016, Han et al. 2022). However, the long-term influence of EMF on root morphology and plant physiology, and their subsequent impacts on drought tolerance remain unclear.

The aim of this study was to investigate the effect of drought on nursery-grown seedlings exposed or nonexposed to various EMF species or soil inoculums. We hypothesized (H1) that the seedlings exposed to EMF or soil inoculums experience a shift in soil microbial community that manifest an increase in seedling transpiration rates during and after drought stress when compared with nonexposed seedlings. We hypothesized (H2) that the effect of the EMF exposure is different depending on the EMF species and this difference is explained by their different hyphal functional traits; EMF with long exploration and hydrophobic hyphae benefit the seedlings more than short and medium exploration and hydrophilic hyphae. We also hypothesized (H3) that EMF and soil inoculums will change the plant biomass distribution (belowground vs aboveground), increase the chlorophyll fluorescence response, and positively affect root morphology 5 months after exposure. Finally, we hypothesized (H4) that these potential changes in root morphology explain the plant’s transpiration response during and after drought stress.

## Materials and methods

### Plant material and growing conditions

In this study, we used 560 1-year-old Scots pine (*Pinus sylvestris* L.) seedlings varying from 15 to 20 cm in height. They were acquired from a tree nursery (Fin Forelia, Röykkä, Finland; https://finforelia.fi) in southern Finland. The seedling roots were washed gently but thoroughly to eliminate any original soil before being transplanted into 0.5-L pots filled with a commercial mixture of coarse white sphagnum peat, dark peat, and sand (pH = 5.9, Seedling substrate W R8494, Kekkilä professional, Vantaa, Finland; https://www.kekkilaprofessional.com/fi) without further fertilization. After this, the seedlings were allowed to recover for 2 weeks in the greenhouse of the University of Helsinki under controlled conditions (relative humidity 35% to 60%, day/night temperature 19/14 °C, radiation 400 W m^−2^ following the daytime light cycle).

### Exposure

We exposed Scots pine seedlings to 10 EMF species commonly found in boreal stands, and to soil inoculums collected from 3 forest stands at Hyytiälä Forestry Field Station (WGS84: N 61°50′43″ E 24°17′13″). One soil inoculum was collected from a fertile unthinned 60-year-old Scots pine-dominated stand (Soil 1), another from a similar Scots pine stand that has been recently thinned and the soil community disturbed by harvesting (Soil 2), and the third from a drier and less fertile unthinned Scots pine stand (Soil 3).

Groups of 40 randomly selected seedlings were exposed to one EMF species each, the strains isolated from boreal Scot pine roots (Table 1). The EMF species were provided by the Microbial Domain Biological Resource Center HAMBI at the University of Helsinki Microbiology Department (https://kotka.luomus.fi/culture/fbcc), where details of each species are described. The strains were grown on Petri dishes on both solid Hagem’s agar media (Stenlid 1985) and on liquid Hagem’s media (same composition but without agar). The pots were first inoculated with biomass obtained from liquid cultures, then twice with 1 cm^2^ plugs of agar media with fungi. All inoculants were placed inside the pots. The nonexposed control pots were amended with similar quantities of Hagem’s media. To test the effects of natural microbial soil communities found in boreal ecosystems, the remaining three groups were inoculated with 1 g (fresh weight) of forest soil inoculum (organic layer). After the EMF or soil inoculums exposure, the grouped seedlings were placed on plastic trays to avoid contamination by water runoff, and their location inside the greenhouse room was changed every other day to avoid possible differences experienced due to varying conditions inside the greenhouse. The colonization success was qualitatively inspected from the potted soil, but no quantitative analysis was made. The seedlings grew under the same controlled greenhouse conditions for 5 months.

**Table 1:** List of EMF species, abbreviations used in the manuscript, database codes (https://kotka.luomus.fi/culture/fbcc), exploration type hyphae and hydrophobicity.

EMF species	Original specimen ID	Exploration type	Hydrophobicity
*Amanita porphyria* (Ap)	FBCC 1398	Medium	Hydrophobic
*Cenococcum geophilum* (Cg)	FBCC 1409	Contact	Hydrophilic
*Laccaria laccata* (Ll)	FBCC 2123	Medium	Hydrophilic
*Lactarius rufus* isolate rufus008 (Lr)	FBCC 1389	Contact	Hydrophilic
*Hyaloscypha variabilis* (Hv)	FBCC2112	Contact	Hydrophilic
*Piloderma olivaceum* (Po)	FBCC 1391	Medium	Hydrophobic
*Rhizopogon roseolus* clone NS202A (Rr)	FBCC 1410	Long	Hydrophobic
*Russula* sp. (R)	FBCC 2133	Contact	Hydrophilic
*Suillus bovinus* DAN (Sb)	FBCC 2054	Long	Hydrophobic
*Suillus variegatus* (Sv)	FBCC 2091	Long	Hydrophobic

Source: based on Agerer (2001), Bogdanova et al. (2023), Peay et al. (2011), Lehto and Zwiazek (2011), Lilleskov et al. (2011), Unestam (1991), and Unestam and Sun (1995).

### Drought treatment and transpiration measurements

The drought experiment took place under controlled greenhouse conditions. For this experiment, we randomly selected 20 seedlings from each exposure group. Ten of the seedlings were placed in the non-drought stress treatment: the seedlings were watered three times a week to field capacity. The remaining 10 seedlings were drought-stressed: watering was fully stopped for 10 days, after which watering resumed for 14 days to evaluate the recovery. To prevent differences in soil moisture content at the start of the experiment, all the seedlings were watered to soil field capacity the day before the drought experiment began.

Transpiration was measured through the potted seedlings’ mass loss. The measurements were carried out between 6 and 8 a.m. every second day, and immediately before and after the driest point on Days 9, 10 and 11. The duration of the drought experiment was decided by observing the number of days it took similar Scots pine seedlings to die under drought stress and by choosing a conservative number of days to guarantee the plants’ survivability. The transpiration measurements began by covering the pots with plastic wrap to prevent water loss through soil evapotranspiration. Then all 10 seedlings of each group were weighed twice, with a 2-hour interval between the measurements, using a digital balance (Precisa 1000c-3000d, accuracy: 0.01 g). Transpiration was calculated based on the water lost during that period. Finally, the plastic wrap was removed from the pots. The transpiration was expressed per unit of dry needle biomass.

Additionally, drought stress in the seedlings was estimated from the needle water potential of three randomly selected individuals from each EMF exposure group in the drought stress and non-drought stress treatment throughout the experiment. The water potential was measured following the transpiration measurements, between 9 and 10 a.m., using a pressure chamber (PMS Model 1505D-EXP, PMS Instrument Company, Albany, OR, USA).

### Plant phenotype analysis, and fine root and biomass analyses

After the recovery phase of the drought treatments, the non-stressed and drought-stressed seedlings were taken to the National Plant Phenotyping Infrastructure (Helsinki Institute of Life Science, University of Helsinki) facilities to measure their chlorophyll fluorescence F_v_/F_m_ and their steady-state NPQ. Chlorophyll fluorescence was measured with a FluorCam pulse amplitude and a modulated system (model FC-800MF, Photon Systems Instruments, Drásov, Czech Republic), and image capture was performed using the quenching protocol (FluorCam 7.0 software, Photon Systems Instruments, Drásov, Czech Republic), as described in Pollari et al. (2022).

After this, root morphology was evaluated to determine the effects of EMF symbiosis on the seedlings’ root architecture. We randomly selected three seedlings from each EMF exposure group and drought treatment, and systematically subsampled a quarter of the soil volume containing each seedling’s root system. The samples were carefully washed, and the root biomass was determined. Another subsample was carefully cleaned under the microscope, scanned (grayscale, 600 d.p.i.), and the images were analyzed using WinRhizo software (Regent Instruments Inc., Québec, Canada) to determine the SRL (Freschet et al. 2021). For the structural analyses, samples from the drought-stressed and normally irrigated seedlings were combined to increase the sample size of each EMF-exposed group. This was possible because no significant differences were observed between the drought-stressed and normally irrigated plants in plant structural traits (drought duration was not long enough and drought intensity was not high enough to cause changes in plant morphology).

Finally, the above- and below-ground biomasses (root, needle and stem) were determined by drying the plant components in an oven at 70 °C until constant mass.

### Data analysis

First, data normality was evaluated using the Shapiro–Wilk test. As the data were not normally distributed, we conducted nonparametric tests for our analyses. We used the Kruskal–Wallis test to test the differences in the effects of exposure to various EMF species and soil inoculums on transpiration rate at Days 10 and 24 (driest point of the experiment and after the recovery period, respectively). Following this test, we performed a pairwise comparison using the Conover and Iman squared ranks test to analyze the differences in transpiration rates between the control, EMF and the soil inoculums. The Conover and Iman test was adjusted using Benjamini–Hochberg correction to correct for the multiple test comparisons. Additionally, the effect of the exploration type and its hydrophobicity was investigated in a similar way. However, the short-distance and contact exploration types were analyzed together because on its own the short exploration type EMF would only have contained one species. Furthermore, the effects of the EMF and the drought treatment on plant biomass, chlorophyll fluorescence response and SRL were tested using the Scheirer–Ray–Hare (SRH) test to determine which variables show significant differences in their responses. The interaction between EMF and drought treatment was removed from the final analysis because it was not significant. After this, the variables that showed significant differences were tested using the Conover and Iman squared ranks test with the Benjamini–Hochberg correction test, to determine significant differences between EMF and forest soil inoculums against the control. The significance level used was *P*-value = 0.05. Finally, to determine the effect of EMF exposure on plant biomass and morphological changes and their relationships with the transpiration response, we analyzed how those variables that showed significant differences with the control in the previous analysis affected the transpiration rate of the drought-stressed plants at the driest point. For this, we plotted the average values of the significant variables and their error bars for each EMF species against the transpiration rate at the driest point. Then the scatter plot distribution was analyzed using the Error-in-Variables regression model. The data analysis was performed using the conover.test (Dinno 2017) and rcompanion (Mangiafico 2023) packages in R version 4.2.2 (R Core Team 2021).

## Results

### Effect of EMF exposure on transpiration rate

We tested the differences in transpiration rate at the start of the drought experiment and found no significant differences between the control seedlings and the seedlings exposed to EMF or soil inoculums (*P*-value = 0.92). We also tested differences in transpiration rate between the drought-stressed and the normally irrigated seedlings within each EMF exposure treatment and found a significantly lower transpiration rate (*P*-value <0.05) in the drought-stressed seedlings at the driest point of the experiment compared with the normally irrigated plants for all exposure treatments (i.e. nonexposed control, exposure to soil inoculums and exposure to EMF species). Furthermore, we observed no differences between drought-stress and non-drought stress treatments after the recovery period, except for the control, and seedlings exposed to *Cenococcum geophilum*, *Russula* sp. or *Suillus variegatus*, which had higher transpiration rates in the non-drought stress treatment compared with their drought-stressed counterparts, whereas seedlings exposed to *Piloderma olivaceum* had a lower transpiration rate in the non-drought stressed compared with the drought-stressed seedlings (see Table S1 available as Supplementary data at *Tree Physiology* Online). Differences between the start of the experiment, the driest point and after recovery for needle water potential were also significant, showing higher water potential at the start of the experiment (see Table S2 available as Supplementary data at *Tree Physiology* Online), which indicates that the seedlings experienced drought stress. However, seedlings exposed to *Rhizopogon roseolus*, Soil 1 or Soil 3 were exceptions presenting no differences in needle water potential between the start of the experiment and after the recovery period (the time series of the non-drought stress treatment is presented in Figure S3, available as Supplementary data at *Tree Physiology* Online).

**Figure 1:**
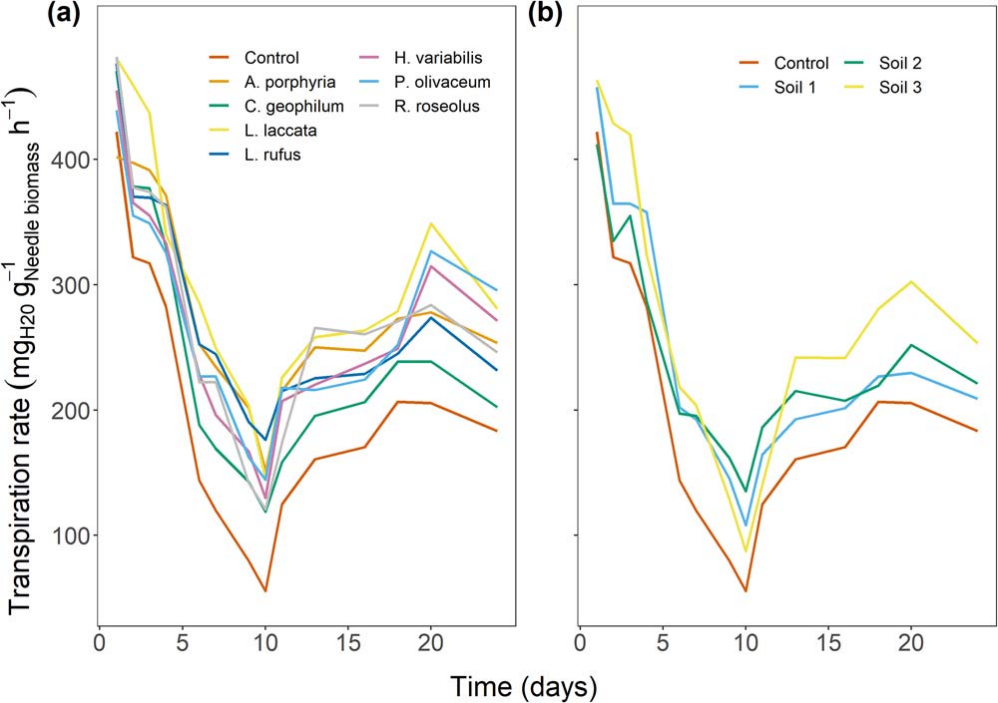
(a) Time series displaying the average transpiration rates of seedlings exposed to EMF species that showed significant differences in transpiration compared with the control under drought-stressed conditions. (b) Time series displaying the average transpiration rates of seedlings exposed to soil inoculums compared with the control under drought-stressed conditions. The transpiration rate declined until it reached the driest point on Day 10; from there, the seedlings recovered after rewatering until the end of the experiment on Day 24.

Overall, the exposure to EMF helped seedlings to maintain higher seedlings transpiration rate compared with the control during the driest point of the drought experiment and after recovery (Table 2). However, only exposure to 7 out of the 10 studied EMF species (*Amanita porphyria*, *Laccaria laccata*, *Lactarius rufus*, *Hyaloscypha variabilis*, *P. olivaceum*, *C. geophilum* and *R. roseolus*) resulted in significantly higher seedling transpiration rates at the driest point of the experiment compared with the control (Figure 1a). After the recovery period, again exposure to seven EMF species (*A. porphyria*, *L. laccata, L. rufus*, *H. variabilis*, *P. olivaceum*, *S. variegatus* and *R. roseolus*) resulted in significant differences in the seedling transpiration rates compared with the control. The control consistently had the lowest transpiration rate throughout the experiment, whereas during the drought treatment, six species consistently exhibited higher transpiration rates compared with the control (*A. porphyria*, *L. laccata*, *L. rufus*, *H. variabilis*, *P. olivaceum* and *R. roseolus*). At the driest point, seedlings exposed to *L. rufus* had the highest transpiration rate, whereas at the end of the recovery period, seedlings exposed to *P. olivaceum* showed the highest transpiration rate. Similarly, the plants exposed to forest soil inoculum generally presented higher transpiration rates than the seedlings in the nonexposed control (Figure 1b). Seedlings exposed to Soil 2 had a significantly higher transpiration rate than the control at the driest point (Table 2) and after the recovery period, whereas the seedlings exposed to Soil 3 had significantly higher transpiration rates than the control after the recovery period.

**Table 2:** Transpiration rate and water potential of drought-stressed plants at the driest point and after the recovery period.

Species	Transpiration rate at the driest point (mgH_2_O g^−1^_needle biomass_ h^−1^)	Transpiration rate after recovery (mgH_2_O g^−1^_needle biomass_ h^−1^)	Needle water potential at the driest point (MPa)	Needle water potential after recovery (MPa)
*Amanita porphyria*	**151.45 ± 13.80**	**245.13 ± 2.76**	−0.96 ± 0.03	−0.52 ± 0.04
*Cenococcum geophilum*	**118.74 ± 19.55**	197.95 ± 6.07	−1.25 ± 0.11	−0.59 ± 0.16
*Laccaria laccata*	**145.10 ± 17.84**	**277.12 ± 9.89**	−1.05 ± 0.07	−0.46 ± 0.04
*Lactarius rufus*	**176.16 ± 4.40**	**224.56 ± 5.85**	−0.97 ± 0.09	−0.50 ± 0.033
*Hyaloscypha variabilis*	**129.98 ± 17.10**	**269.36 ± 13.31**	−1.13 ± 0.12	−0.51 ± 0.03
*Piloderma olivaceum*	**144.42 ± 12.22**	**282.11 ± 8.22**	−1.10 ± 0.02	−0.53 ± 0.04
*Rhizopogon roseolus*	**120.67 ± 13.26**	**240.13 ± 8.03**	−1.16 ± 0.09	−0.51 ± 0.10
*Russula* sp.	98.88 ± 22.21	176.98 ± 14.54	−1.11 ± 0.20	−0.63 ± 0.08
*Suillus bovinus*	106.14 ± 13.63^a^	199.78 ± 8.56	−1.07 ± 0.10	−0.51 ± 0.06
*Suillus variegatus*	64.98 ± 9.97	**206.28 ± 6.13**	−1.21 ± 0.24	−0.60 ± 0.03
Soil 1	108.01 ± 8.92^a^	198.28 ± 4.95	−1.10 ± 0.16	−0.44 ± 0.05
Soil 2	**135.20 ± 14.16**	**217.09 ± 5.98**	−1.03 ± 0.20	−0.51 ± 0.05
Soil 3	87.29 ± 11.87	**244.66 ± 3.38**	−1.08 ± 0.20	−0.53 ± 0.01
Control	55.56 ± 6.24	181.99 ± 6.24	−1.30 ± 0.28	−0.62 ± 0.08

Note: the data are presented as the mean ± standard error (*n* = 10). The transpiration rate results that show significant differences against the control are marked in bold (*P*-values < 0.05).

^a^Tendency to significance (0.05 < *P*-value < 0.1).

The pairwise comparison between EMF species (Table 3) showed that seedlings exposed to *S. variegatus* had significantly lower transpiration rates at the driest point of the experiment than seedlings exposed to other EMF species, except for *Russula* sp. and *Suillus bovinus* which had no statistical difference from *S. variegatus*. Additionally, seedlings exposed to *L. rufus* had significantly higher transpiration rates when compared with seedlings exposed to *H. variabilis*, *R. roseolus*, *Russula* sp. or both *Suillus* species. After recovery, seedlings exposed to *A. porphyria*, *L. laccata*, *L. rufus*, *H. variabilis*, *P. olivaceum* or *R. roseolus* had significantly higher transpiration rates than seedlings exposed to *Russula* sp. or *Suillus* species.

**Table 3:** Pairwise comparison of seedling transpiration responses at the driest point and after recovery between seedlings exposed to EMF species. Ap: *A. porphyria*, Cg: *C. geophilum*, Lc: *L. laccata*, Lr: *L. rufus*, Hv: *H. variabilis*, Po: *P. olivaceum*, Rr: *R. roseolus*, R: *Russula* sp., Sb: *S. bovinus*, Sv: *S. variegatus*.

Transpiration rate at the dries point											
Species	Control	Ap	Cg	Ll	Lr	Hv	Po	R	Rr	Sb	Sv
Control		**<0.01**	**<0.01**	**<0.01**	**<0.01**	**<0.01**	**<0.01**	0.06	**<0.01**	0.06	0.76
Ap			0.21	0.96	0.21	0.47	0.88	**0.05**	0.21	0.06	**<0.01**
Cg				0.22	**0.01**	0.61	0.28	0.5	0.99	0.53	**0.02**
Ll					0.18	0.5	0.92	**0.05**	0.22	0.06	**<0.01**
Lr						**0.04**	0.14	**<0.01**	**0.01**	**<0.01**	**<0.01**
Hv							0.55	0.22	0.61	0.26	**0.01**
Po								0.07	0.28	0.09	**<0.01**
R									0.5	0.95	0.13
Rr										0.53	**0.02**
Sb											0.1
Sv											
**Transpiration rate after recovery**											
**Species**	**Control**	**Ap**	**Cg**	**Ll**	**Lr**	**Hv**	**Po**	**R**	**Rr**	**Sb**	**Sv**
Control		**<0.01**	0.22	**<0.01**	**<0.01**	**<0.01**	**<0.01**	0.71	**<0.01**	0.10	**0.02**
Ap			**<0.01**	**0.03**	**0.03**	0.23	**0.01**	**<0.01**	0.56	**<0.01**	**<0.01**
Cg				**<0.01**	**<0.01**	**<0.01**	**<0.01**	0.38	**<0.01**	0.7	0.31
Ll					**<0.01**	0.35	0.54	**<0.01**	**0.01**	**<0.01**	**<0.01**
Lr						**<0.01**	**<0.01**	**<0.01**	0.12	**0.01**	**0.05**
Hv							0.12	**<0.01**	0.08	**<0.01**	**<0.01**
Po								**<0.01**	**<0.01**	**<0.01**	**<0.01**
R									**<0.01**	0.22	0.06
Rr										**<0.01**	**<0.01**
Sb											0.54
Sv											

Note: significant differences in bold (*P*-value ≤0.05).

**Figure 2:**
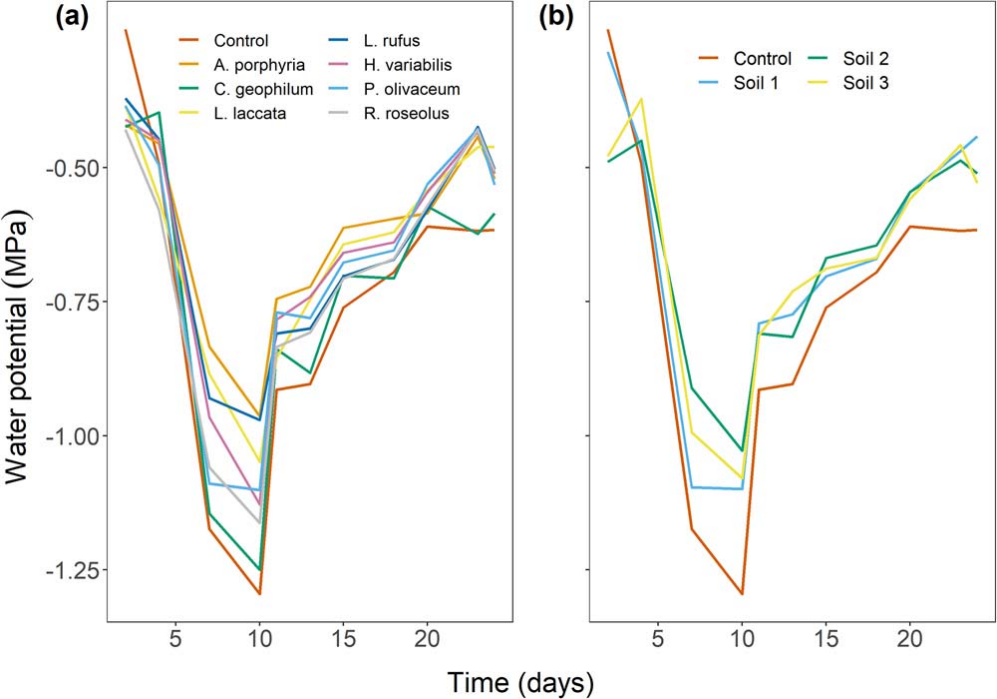
(a) Time series displaying the average needle water potentials of seedlings exposed to EMF species that showed significant differences in transpiration compared with the control under drought-stressed conditions. (b) Time series displaying the average needle water potentials of seedlings exposed to soil inoculums compared with the control under drought-stressed conditions.

The needle water potential displayed similar trends as the transpiration rate, with the control presenting higher levels of drought stress than seedlings exposed to EMF or soil inoculum, indicated by the lower values of needle water potential (Figure 2a and b). However, the differences were not statistically significant either at the driest point or after recovery when comparing exposed seedlings with the control.

**Figure 3:**
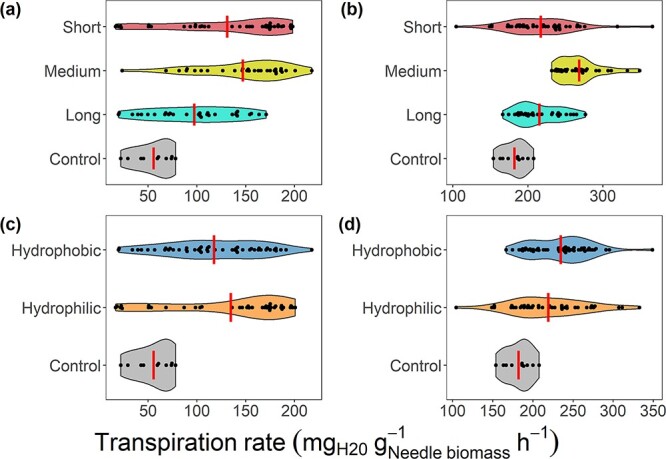
Mean transpiration rate of the control and (a) EMF exposed seedlings grouped by exploration type at the driest point, (b) EMF exposed seedlings grouped by exploration type after recovery, (c) EMF exposed seedlings grouped by hydrophobicity at the driest point and (d) EMF exposed seedlings grouped by hydrophobicity after recovery. The error bars represent the standard error (control: *n* = 10; long and medium: *n* = 30; short: *n* = 40; hydrophobicity: *n* = 50 per-treatment).

As we found differences in transpiration rates in EMF exposed seedlings, we analyzed their functional traits to determine if exploration type and hydrophobicity may explain the seedlings, response to drought. At the driest point, seedlings exposed to short- or medium-distance exploration type EMF had higher transpiration rates than long-distance exploration type (*P*-value <  0.01) (Figure 3a), and there were no significant differences in transpiration rates between seedlings exposed to short- and medium-distance exploration EMF (*P*-value = 0.24). After recovery, seedlings exposed to medium-distance exploration type EMF had higher transpiration rate than seedlings exposed to short- or long-distance exploration type EMF (*P*-value < 0.01) (Figure 3b), whereas no significant differences were observed between seedlings exposed to short- and long-distance exploration type EMF (*P*-value = 0.94). In addition, seedlings exposed to hydrophilic EMF had marginally higher transpiration rates than seedlings exposed to hydrophobic EMF at the driest point (*P*-value = 0.06) (Figure 3c), whereas their differences after recovery were not significant (*P*-value = 0.36) (Figure 3d).

### Effect of EMF exposure on root morphology, chlorophyll fluorescence and biomass allocation

The effect of EMF and soil inoculum exposure on plant morphology and physiology was explored by jointly analyzing the effects of the drought-stressed and non-drought stressed plants and EMF species exposure (Table 4). Seedling exposure to different EMF species increased the stem biomass and reduced the SRL. Additionally, drought stress significantly decreased seedling total biomass and root biomass. Ectomycorrhizal fungi exploration type and hydrophobicity affected F_v_/F_m_ with short exploration type EMF having lower values than both the control (*P*-value = 0.02) and long-distance exploration type EMF (*P*-value = 0.02). We found significant lower values of F_v_/F_m_ between the control and seedlings exposed to hydrophilic EMF (*P*-value = 0.04).

**Table 4:** Summary table showing the biomass, chlorophyll fluorescence and root morphology results.

Species	Total biomass (g)^a^	Needle biomass (g)	Stem biomass (g)	Root biomass (g)^a^	Maximum quantum yield (F_v_/F_m_)	Steady-state NPQ	SRL (m.g^−1^)^a^
Control	11.55 ± 0.32	3.44 ± 0.11	1.78 ± 0.09	5.89 ± 0.40	0.83 ± 0.00	2.44 ± 0.08	9.79 ± 0.70
*Amanita porphyria*	11.15 ± 0.47	3.58 ± 0.11	1.97 ± 0.09	5.50 ± 0.32	0.81 ± 0.02	2.43 ± 0.08	6.82 ± 1.32
*Cenococcum geophilum*	11.05 ± 0.72	3.37 ± 0.13	1.84 ± 0.11	5.75 ± 0.52	0.79 ± 0.01	2.66 ± 0.12	6.27 ± 0.90
*Laccaria laccata*	9.89 ± 0.80	3.48 ± 0.14	2.16 ± 0.15	4.75 ± 0.32	0.81 ± 0.01	2.54 ± 0.10	9.59 ± 1.22
*Lactarius rufus*	11.49 ± 0.87	3.68 ± 0.14	2.19 ± 0.09	5.91 ± 0.71	0.81 ± 0.01	2.51 ± 0.07	8.11 ± 1.04
*Hyaloscypha variabilis*	11.64 ± 0.82	3.82 ± 0.17	2.12 ± 0.09	5.77 ± 0.67	0.81 ± 0.01	2.61 ± 0.10	10.50 ± 1.50
*Piloderma olivaceum*	12.17 ± 0.78	3.67 ± 0.17	2.06 ± 0.09	6.09 ± 0.45	0.81 ± 0.01	2.64 ± 0.12	10.12 ± 0.79
*Rhizopogon roseolus*	12.05 ± 0.72	3.46 ± 0.16	2.06 ± 0.10	6.28 ± 0.51	0.82 ± 0.01	2.51 ± 0.11	7.72 ± 0.51
*Russula* sp*.*	11.19 ± 1.27	3.49 ± 0.13	1.94 ± 0.10	5.97 ± 0.93	0.80 ± 0.01	2.61 ± 0.13	7.67 ± 1.31
*Suillus bovinus*	11.47 ± 0.66	3.76 ± 0.13	2.08 ± 0.08	5.61 ± 0.60	0.81 ± 0.01	2.59 ± 0.10	6.88 ± 0.80
*Suillus variegatus*	10.36 ± 0.52	3.69 ± 0.15	2.00 ± 0.10	4.72 ± 0.50	0.84 ± 0.00	2.37 ± 0.07	11.65 ± 1.89
Soil 1	10.71 ± 0.66	3.58 ± 0.19	1.80 ± 0.10	5.06 ± 0.44	0.82 ± 0.01	2.57 ± 0.11	11.61 ± 1.12
Soil 2	12.05 ± 0.76	3.74 ± 0.16	1.90 ± 0.10	6.41 ± 0.43	0.81 ± 0.01	2.75 ± 0.15	9.69 ± 1.03
Soil 3	10.94 ± 0.90	3.46 ± 0.13	2.08 ± 0.12	5.58 ± 0.70	0.81 ± 0.01	2.56 ± 0.13	10.38 ± 1.32
EMF species	NS	NS	**0.05**	NS	NS	NS	**0.02**
Drought treatment	**0.05**	0.07	NS	**0.02**	NS	NS	NS
Exploration type^b^	NS	NS	NS	NS	**<0.01**	NS	NS
Hydrophobicity^b^	NS	NS	0.07	NS	**0.02**	NS	0.09

Note: the data are presented as the mean ± standard error (*n* = 20) (^a^*n* = 6). The significance of EMF species and the drought treatment was tested with the SRH test. ^b^Kruskal–Wallis test was used because of imbalanced data. Significance values *P* < 0.05 (NS = not significant).

**Figure 4:**
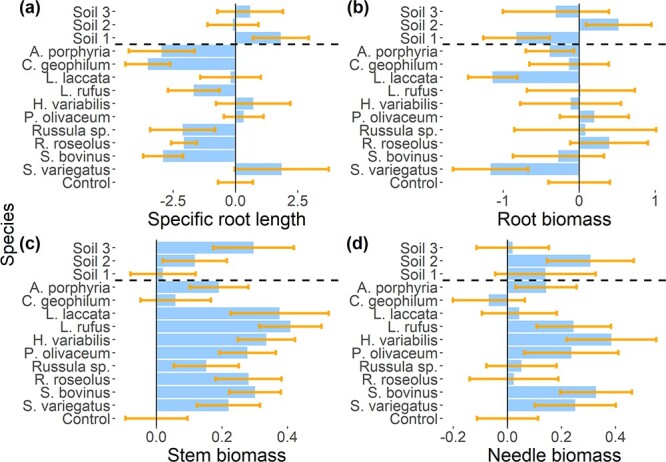
Analysis of (a) SRL, (b) root biomass, (c) stem biomass and (d) needle biomass by EMF species and soil inoculum. The bars represent the exposure treatments mean with the control mean subtracted. The error bars represent the standard error (SRL and root biomass: *n* = 6; stem and needle biomass: *n* = 20). The horizontal dashed line separates EMF species and soil inoculums.

**Figure 5:**
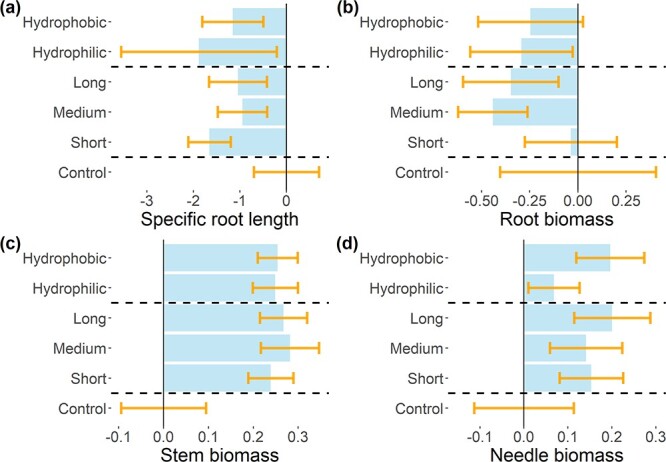
Analysis of (a) SRL, (b) root biomass, (c) stem biomass and (d) needle biomass by EMF hydrophobicity and exploration type. The bars represent the mean values with the control mean subtracted. The error bars represent the standard error (control: *n* = 20; long and medium: *n* = 60; short: *n* = 80; hydrophobicity: *n* = 100). The horizontal dashed line separates EMF hydrophobicity and exploration type.

Following the previous results, we analyzed the differences in stem biomass and SRL caused by EMF exposure. Although the SRH test showed differences between the EMF species in stem biomass and SRL, the post hoc Conover and Iman test failed to find significant differences between each EMF exposed seedlings and the control. In Figure 4, we present the average SRL as well as stem, root and needle biomass of the control and exposed seedlings. Furthermore, in Figure 5, we can observe no significant differences in seedling biomass or SRL when grouping the seedlings exposed to EMF by their exploration type or hydrophobicity.

### Relationship between plant morphology and transpiration rate

**Figure 6:**
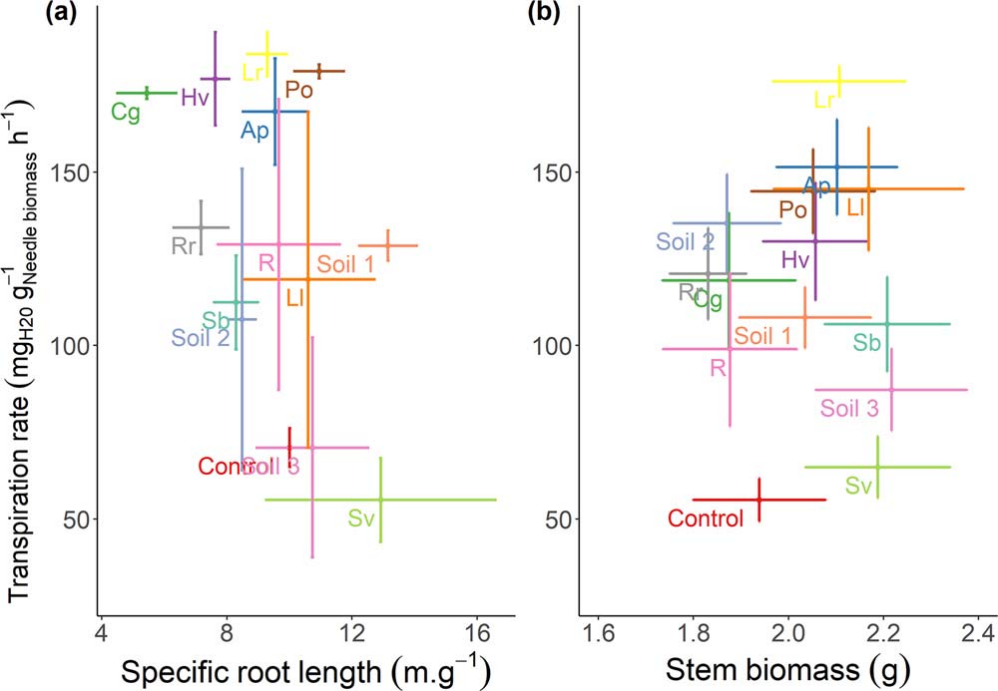
Scatter plot showing the relationship between (a) transpiration rate and stem biomass by EMF species, and (b) transpiration rate and SRL by EMF species. The error bars represent the standard error (SRL *n* = 3; stem biomass *n* = 10). Ap: *A. porphyria*, Cg: *C. geophilum*, Lc: *L. laccata*, Lr: *L. rufus*, Hv: *H. variabilis*, Po: *P. olivaceum*, Rr: *R. roseolus*, R: *Russula* sp., Sb: *S. bovinus*, Sv: *S. variegatus*, Soil 1: unthinned forest soil, Soil 2: thinned forest soil, Soil 3: dry forest soil.

Because these SRL and stem biomass showed significant differences between the EMF species, we tested if they can explain the observed difference in transpiration rate across exposed seedlings. Neither changes in stem biomass nor changes in SRL because of EMF exposure could explain the differences in transpiration rate across the exposed seedlings at the driest point (Figure 6a and b; *R* = 0.01; *P*-value = 0.97 and *R* = 0.45; *P*-value = 0.10, respectively). Additionally, we also found no correlation between stem biomass and SRL after recovery (*R* = 0.34; *P*-value = 0.24 and *R* = 0.08; *P*-value = 0.8, respectively).

## Discussion

Exposure to EMF had an overall positive effect on the transpiration responses of the seedlings under drought conditions with some species-specific differences. This supports our first hypothesis that seedlings exposed to EMF maintain a higher transpiration rate than nonexposed seedlings under dry conditions and present faster recovery once watering is resumed. We also hypothesize that the exposure to soil inoculum stimulates the formation of natural soil microbial community in the experimental soils, and would enable seedlings to maintain higher transpiration rate under dry conditions when compared with the nonexposed seedlings. Interestingly, we found contrasting results in soil inoculum-exposed plants between different soil origins. This suggests that soil inoculum exposure has the potential to benefit nursery-raised seedlings. However, the effects are highly variable, highlighting the need for additional studies with more repetitions to confirm our findings.

At the species level, *R. roseolus*-exposed seedlings exhibited high transpiration rates throughout the drought experiment, which is consistent with previous studies indicating drought resistance of the *Rhizopogon* genus and its potential ability to mitigate drought stress for the host plant (Parke et al. 1983, Coleman et al. 1989, Castaño et al. 2023). Similarly, species in the *Cenococcum* genus were found to be drought tolerant (Coleman et al. 1989, Nilsen et al. 1998), which may explain the positive effect they had on the seedlings’ response to drought in this study. Also, in our study, seedlings exposed to *L. laccata* maintained higher transpiration rates compared with nonexposed seedlings. This contrasts with the findings of Parke et al. (1983), who observed no differences in transpiration response with the same species. Similarly, Boyle and Hellenbrand (1991) observed lower plant growth in association with *L. laccata* under drought conditions relative to other EMF species, although still higher than the non-inoculated control. The differences between our results and previous studies done at the genus level may be attributed to interspecific differences within a genus, as emphasized by Coleman et al. (1989). In their investigation, Coleman et al. studied the differences in drought tolerance among various EMF species, including eight *Suillus* species, revealing variations in drought responses across these *Suillus* species, supporting the observed differences in transpiration response between *S. variegatus* and *S. bovinus* in our study.

In this study, the EMF species had different functional traits, considered to influence fungal foraging ability (Unestam 1991, Lehto and Zwiazek 2011). Given the observed differences in seedling response to drought stress, we investigated whether these differences could be attributed to their exploitation type and hydrophobicity. Seedlings associated with medium-distance exploration type EMF performed better under drought stress than seedlings associated with short- and long-distance exploration type. This result differs from previous studies that described higher benefits of long-distance exploration EMF under drought stress when compared with other exploration types (Agerer 2001, Garcia de Jalon et al. 2020, Castaño et al. 2023). However, it is possible that in nature, long-distance exploration may be more effective, given the opportunity to explore various soil layers. In contrast, our study utilized smaller pots, potentially creating conditions favoring medium-distance exploration, and it is possible that the performance of EMF exploration type may be aligned to water distribution patterns. Ectomycorrhizal fungi hydrophobicity did not seem to affect the transpiration response of the exposed seedlings as the best-performing seedlings were associated with either hydrophobic or hydrophilic EMF. The lack of effect of hydrophobicity and contrasting exploration type findings are consistent with questioning of the current classification (Tedersoo et al. 2012, Jörgensen et al. 2023). While more studies are needed to fully understand the utility of the current classification, our results do not align with our second hypothesis and more importantly challenge the functional trait approach and call for a more species-specific assessment of EMF impact on plant drought tolerance.

Ectomycorrhizal fungi exposure is known to change the host plant physiological and morphological attributes (Sun et al. 2010, Heinonsalo et al. 2015), and these induced changes could be responsible for the observed differences in drought response. However, in our study, neither EMF nor soil inoculum exposure impacted plant chlorophyll fluorescence response (F_v_/F_m_ and NPQ) 5 months after the EMF exposure. The fluorescence response of the seedlings was measured after recovery, i.e. we were unable to test the short-term effects of drought on the photosynthetic apparatus. Although there was no effect of EMF exposure on the Photosystem II after recovery, several studies have shown the negative effects of drought on the fluorescence response (Chen et al. 2022, Cousins et al. 2002, Sebastiana et al. 2018), and the ability of the exposed seedlings to maintain higher levels of transpiration under dry conditions suggests an increase in water uptake capacity and potentially higher levels of photosynthesis, thereby improving plant survival under drought stress. Similarly, Heinonsalo et al. (2015) found that the presence of EMF did not affect plant chlorophyll fluorescence and, instead, the plant photosynthetic process was affected by changes in nitrogen allocations and water economy. Accordingly, previous reports of increased photosynthetic activity because of EMF (Colpaert et al. 1996, Nehls 2008) could be attributed to enhanced root water and nutrient uptake capacity provided by the EMF hyphae.

Ectomycorrhizal fungi exposure did not affect stem biomass or SRL. These findings are in line with Rincon et al. (2001), who found no significant growth differences in *Pinus pinea* seedlings exposed to seven EMF species. However, several other previous studies reported an increase in overall biomass production in plants inoculated with EMF (Dosskey et al. 1992, Kipfer et al. 2012). These studies differed from ours in the experimental design, utilizing younger seedlings and featuring variations in soil moisture and fertilization. Our results, together with the results by Rincon et al. (2001), could also imply that potential increases in carbon assimilation in the exposed seedlings would be allocated to the EMF growth instead of the growth of the host plant (Colpaert et al. 1996, Heinonsalo et al. 2004, 2010). However, this was beyond the scope of our study. It should also be noted that the number of replicates measured for root characteristics was moderate and the experimental period was probably not long enough to cause detectable changes in biomass distribution. To summarize, our results did not support the third hypothesis as the EMF exposure did not significantly affect either the plant biomass distribution (belowground vs aboveground), plant chlorophyll fluorescence or root morphology.

Our findings did not support that potential changes in root morphology may explain the transpiration response of seedlings to drought stress (H4), as changes in root morphology were not detected and could therefore not explain the plant transpiration response during and after the drought stress. Additionally, we observed that the potential changes in stem allocation caused by EMF had no effect on the transpiration rate response under drought conditions. Our findings on SRL contradict previous results showing an increase in SRL in plants exposed to drought conditions (Olmo et al. 2014). One possible explanation could be the low number of replicates in measuring root structure for each treatment. Additionally, variations in colonization success may have impacted fungal biomass, potentially masking any root morphological changes induced by EMF and consequently influencing root size and overall biomass (Colpaert et al. 1992, Ekblad et al. 1995). However, despite these potential limitations, the significantly higher transpiration responses exhibited by most seedlings exposed to EMF species compared with the control suggests that colonization was generally successful. Therefore, we conclude that the observed effects in this study can be attributed to EMF species exposure.

Other factors that were not analyzed in this study may have played a role in the transpiration responses of the host plants under stressed conditions, such as soil microbial community. This study was not carried out in sterile soil conditions and aimed to mimic commercial tree nursery conditions, meaning that exposing the seedlings to selected EMF resulted, even with best success, in a partial change in root and soil fungal community. Microbial communities may contribute to climate tolerance and, in general, introduction of new microbial taxa to the soil may change the microbial community interactions and affect plant environmental responses (Allsup et al. 2023, Mawarda et al. 2020, Liu et al. 2021). More experimentation on axenic conditions is needed to verify individual EMF species contributions to the host plant’s drought-stress tolerance. However, in such axenic studies, the complex interactions between soil flora and fauna found in natural forest stands would be lost. Consequently, we believe that the observed differences highlight the importance of the studied fungal associates with soil community on plant drought responses. Despite the need for further study under field conditions, our results demonstrate that exposure of nursery-raised seedlings to selected EMF increases plant drought tolerance. This finding suggests a potential practical strategy in nursery plant production for forest regeneration activities.

In conclusion, exposure with most EMF species and soil inoculums increases the transpiration rate of nursery-raised Scots pine seedlings under drought conditions and post-recovery when compared with nonexposed seedlings. Ectomycorrhizal fungi functional traits do not seem to explain the species-specific differences in the seedling drought responses and thus hints that the current EMF classification based on exploration type and hydrophobicity might not systematically be a reliable predictor for the response of exposed seedlings to drought. Ectomycorrhizal fungi exposure had no discernible impact on plant morphology. Nevertheless, due to the moderate sample size, the effects of various EMF and soil inoculums on SRL and biomass distribution should continue to be explored. These findings imply that exposing seedlings to EMF could be a practical strategy to enhance drought tolerance during the production of plants in nurseries, which may be a valuable aspect for forest regeneration, particularly in regions or times where drought conditions are a concern.

## Supplementary Material

Supporting_information_tpae029

## Data Availability

The data supporting the findings in this study are available from https://doi.org/10.5281/zenodo.10100580.

## References

[ref1] Aaltonen H , Lindén A, Heinonsalo J, Biasi C, Pumpanen J (2016) Effects of prolonged drought stress on Scots pine seedling carbon allocation. Tree Physiol 37:418–427. 10.1093/treephys/tpw119. 27974653

[ref2] Agerer R (2001) Exploration types of ectomycorrhizae: a proposal to classify ectomycorrhizal mycelial systems according to their patterns of differentiation and putative ecological importance. Mycorrhiza 11:107–114. 10.1007/s005720100108.

[ref3] Allsup CM , George I, Lankau RA (2023) Shifting microbial communities can enhance tree tolerance to changing climates. Science 380:835–840. 10.1126/science.adf2027. 37228219

[ref4] Alvarez M , Huygens D, Fernandez C, Gacitua Y, Olivares E, Saavedra I, Alberdi M, Valenzuela E (2009) Effect of ectomycorrhizal colonization and drought on reactive oxygen species metabolism of *Nothofagus dombeyi* roots. Tree Physiol 29:1047–1057. 10.1093/treephys/tpp038. 19483186

[ref5] Aryal P , Meiners SJ, Carlsward BS (2020) Ectomycorrhizae determine chestnut seedling growth and drought response. Agrofor Syst 95:1251–1260. 10.1007/s10457-020-00488-4.

[ref6] Baskaran P , Hyvonen R, Berglund SL, Clemmensen KE, Agren GI, Lindahl BD, Manzoni S (2017) Modelling the influence of ectomycorrhizal decomposition on plant nutrition and soil carbon sequestration in boreal forest ecosystems. New Phytol 213:1452–1465. 10.1111/nph.14213. 27748949

[ref7] Beniwal RS , Langenfeld-Heyser R, Polle A (2010) Ectomycorrhiza and hydrogel protect hybrid poplar from deficit and unravel plastic responses of xylem anatomy. Environ Exp Bot 69:189–197. 10.1016/j.envexpbot.2010.02.005.

[ref8] Bödeker ITM , Clemmensen KE, de Boer W, Martin F, Olson Å, Lindahl BD (2014) Ectomycorrhizal *Cortinarius* species participate in enzymatic oxidation of humus in northern forest ecosystems. New Phytol 203:245–256. 10.1111/nph.12791. 24725281

[ref9] Bogdanova O , Kothe E, Krause K (2023) Ectomycorrhizal community shifts at a former uranium mining site. Fungi 9:483. 10.3390/jof9040483. PMC10144560 37108937

[ref10] Boyle CD , Hellenbrand KE (1991) Assessment of the effect of mycorrhizal fungi on drought tolerance of conifer seedlings. Can J Bot 69:1764–1771. 10.1139/b91-224.

[ref11] Caminero L , Génova M, Camarero JJ, Sánchez-Salguero R (2018) Growth responses to climate and drought at the southernmost European limit of Mediterranean *Pinus pinaster* forests. Dendrochronologia 48:20–29. 10.1016/j.dendro.2018.01.006.

[ref12] Carnicer J , Coll M, Ninyerola M, Pons X, Sánchez G, Peñuelas J (2011) Widespread crown condition decline, food web disruption, and amplified tree mortality with increased climate change-type drought. Proc Natl Acad Sci 108:1474–1478. 10.1073/pnas.1010070108. 21220333 PMC3029725

[ref13] Castaño C , Suarez-Vidal E, Zas R, Bonet JA, Oliva J, Sampedro L (2023) Ectomycorrhizal fungi with hydrophobic mycelia and rhizomorphs dominate in young pine trees surviving experimental drought stress. Soil Biol Biochem 178:108932. 10.1016/j.soilbio.2022.108932.

[ref14] Castro J , Zamora R, Hodar JA, Gomez JM (2004) Seedling establishment of a boreal tree species (*Pinus sylvestris*) at its southernmost distribution limit: consequences of being in a marginal Mediterranean habitat. J Ecol 92:266–277. 10.1111/j.0022-0477.2004.00870.x.

[ref15] Chakravarty P , Unestam T (1987) Differential influence of ectomycorrhizae on plant growth and disease resistance in *Pinus sylvestris* seedlings. J Phytopathol 120:97–192.

[ref16] Chalot M , Brun A (1998) Physiology of organic nitrogen acquisition by ectomycorrhizal fungi and ectomycorrhizas. FEMS Microbiol Rev 22:21–44. 10.1111/j.1574-6976.1998.tb00359.x. 9640645

[ref18] Chen Z , Liu Z, Han S, Jiang H, Xu S, Zhao H, Ren S (2022) Using the diurnal variation characteristics of effective quantum yield of PSII photochemistry for drought stress detection in maize. Ecol Indic 138:108842. 10.1016/j.ecolind.2022.108842.

[ref19] Coleman MD , Bledsoe CS, Lopushinsky W (1989) Pure culture response of ectomycorrhizal fungi to imposed water stress. Can J Bot 67:29–39. 10.1139/b89-005.

[ref20] Colpaert JV , Van Assche JA, Luijtens K (1992) The growth of the extramatrical mycelium of ectomycorrhizal fungi and the growth response of *Pinus sylvestris* L. New Phytol 120:127–135. 10.1111/j.1469-8137.1992.tb01065.x.

[ref21] Colpaert JV , Van Laere A, Van Assche JA (1996) Carbon and nitrogen allocation in ectomycorrhizal and non-mycorrhizal *Pinus sylvestris* L. seedlings. Tree Physiol 16:787–793. 14871686 10.1093/treephys/16.9.787

[ref22] Cousins AB , Adam NR, Wall GW, Kimball BA, Pinter P, Ottman MJ, Leavitt SW, Webber AN (2002) Photosystem II energy use, non-photochemical quenching and the xanthophyll cycle in *Sorghum bicolor* grown under drought and free-air CO_2_ enrichment (FACE) conditions. Plant Cell Environ 25:1551–1559.

[ref23] Dinno A (2017) _conover.test: Conover-Iman Test of multiple comparisons using rank sums_. R package version 1.1.5. https://CRAN.R-project.org/package=conover.test.

[ref24] Dosskey MG , Liderman RG, Boersma L (1992) Comparison of biomass allocation in ectomycorrhizal and nonmycorrhizal Douglas fir seedlings of similar nutrition and overall size. Plant Soil 142:147–150. 10.1007/BF00010185.

[ref25] Duan X , Neuman DS, Reiber JM, Green CD, Saxton AM, Augé RM (1996) Mycorrhizal influence on hydraulic and hormonal factors implicated in the control of stomatal conductance during drought. J Exp Bot 47:1541–1550. 10.1093/jxb/47.10.1541.

[ref26] Ekblad A , Wallander H, Carlsson R, Huss-Danell K (1995) Fungal biomass in roots and extramatrical mycelium in relation to macronutrients and plant biomass of ectomycorrhizal *Pinus sylvestris* and *Alnus incana*. New Phytol 131:411–563. 33863123 10.1111/j.1469-8137.1995.tb03081.x

[ref27] Emerson R (1958) The quantum yield of photosynthesis. Annu Rev Plant Physiol 9:1–24. 10.1146/annurev.pp.09.060158.000245.

[ref28] Evans JR (1987) The dependence of quantum yield on wavelength and growth irradiance. Aust J Plant Physiol 14:69–79.

[ref29] Freschet GT , Pagès L, Iversen CM et al. (2021) A starting guide to root ecology: strengthening ecological concepts and standardising root classification, sampling, processing and trait measurements. New Phytol 232:973–1122. 10.1111/nph.17572. 34608637 PMC8518129

[ref30] Garcia de Jalon L , Limousin JM, Richard F, Gessler A, Peter M, Hattenschwiler S, Milcu A (2020) Microhabitat and ectomycorrhizal effects on the establishment, growth and survival of *Quercus ilex* L. seedlings under drought. PloS One 15:e0229807. 10.1371/journal.pone.0229807. 32502167 PMC7274372

[ref31] Gehring CA , Swaty R, Deckert R (2017) Mycorrhizas, drought, and host-plant mortality. In: Johnson NC, Gehring C, Jansa J, editors. Mycorrhizal mediation of soil. Holland: Elsevier, pp 279–298.

[ref32] Grossnickle SC , MacDonald JE (2018) Why seedlings grow: influence of plant attributes. New For 49:1–34. 10.1007/s11056-017-9606-4.

[ref33] Han X , Li Y, Li Y, Du X, Li B, Li Q (2022) Soil inoculum identity and rate jointly steer microbiomes and plant communities in the field. ISME Commun 2:59. 10.1038/s43705-022-00144-1. 37938291 PMC9723724

[ref34] Heinonsalo J , Hurme K-R, Sen R (2004) Recent 14C-labeled assimilate allocation to Scots pine seedling root and mycorrhizosphere compartments developed on reconstructed podzol humus, E- and B- mineral horizons. Plant Soil 259:111–121. 10.1023/B:PLSO.0000020939.64205.c4.

[ref36] Heinonsalo J , Pumpanen J, Rasilo T, Hurme K-R, Ilvesniemi H (2010) Carbon partitioning in ectomycorrhizal Scots pine seedlings. Soil Biol Biochem 42:1614–1623. 10.1016/j.soilbio.2010.06.003.

[ref35] Heinonsalo J , Juurola E, Linden A, Pumpanen J (2015) Ectomycorrhizal fungi affect Scots pine photosynthesis through nitrogen and water economy, not only through increased carbon demand. Environ Exp Bot 109:103–112. 10.1016/j.envexpbot.2014.08.008.

[ref37] Helenius P , Luoranen J, Rikala R, Leinonen K (2002) Effect of drought on growth and mortality of actively growing Norway spruce container seedlings planted in summer. Scand J For Res 17:218–224. 10.1080/028275802753742882.

[ref38] Horton P , Ruban AV, Walters RG (1994) Regulation of light harvesting in green plants. Plant Physiol 106:415–420. 10.1104/pp.106.2.415. 12232338 PMC159545

[ref39] Irvine J , Perks MP, Magnani F, Grace J (1998) The response of *Pinus sylvestris* to drought: stomatal control of transpiration and hydraulic conductance. Tree Physiol 18:393–402. 10.1093/treephys/18.6.393. 12651364

[ref40] Jinfei Y , Xiaobing Z, Benfeng Y, Yonggang L, Yuanming Z (2020) Species-dependent responses of root growth of herbaceous plants to snow cover changes in a temperate desert, Northwest China. Plant Soil 459:249–260.

[ref41] Jörgensen K , Clemmensen KE, Wallander H, Lindahl BD (2023) Do ectomycorrhizal exploration types reflect mycelial foraging strategies? New Phytol 237:576–584. 10.1111/nph.18566. 36271619 PMC10098516

[ref42] Junker LV , Kleiber A, Jansen K et al. (2017) Variation in short-term and long term responses of photosynthesis and isoprenoid-mediated photoprotection to soil water availability in four Douglas-fir provenances. Sci Rep 7:40145. 10.1038/srep40145. 28071755 PMC5223217

[ref43] Kipfer T , Wohlgemuth T, Van der Heijden MGA, Ghazoul J, Egli S (2012) Growth response of drought-stressed *Pinus sylvestris* seedlings to single- and multi-species inoculation with ectomycorrhizal fungi. PloS One 7:e35275. 10.1371/journal.pone.0035275 22496914 PMC3320646

[ref44] Lehto T (1992) Mycorrhizas and drought resistance of *Picea sitchensis* (Bong.) Carr. New Phytol 122:571–783.

[ref45] Lehto T , Zwiazek J (2011) Ectomycorrhizas and water relations of trees: a review. Mycorrhiza 21:71–90. 10.1007/s00572-010-0348-9. 21140277

[ref46] Lilleskov EA , Hobbie EA, Horton TR (2011) Conservation of ectomycorrhizal fungi: exploring the linkages between functional and taxonomic responses to anthropogenic N deposition. Fungal Ecol 4:174–183. 10.1016/j.funeco.2010.09.008.

[ref47] Liu H , Qiu Z, Ye J, Verma JP, Li J, Singh BK (2021) Effective colonisation by a bacterial synthetic community promotes plant growth and alters soil microbial community. J Sustain Agric Environ 1:30–42. 10.1002/sae2.12008.

[ref48] Lu C , Zhang J (1999) Effects of water stress on photosystem II photochemistry and its thermostability in wheat plants. J Exp Bot. 50(336): 1199–1206 10.1093/jxb/50.336.1199.

[ref49] Luo ZB , Li K, Gai Y, Göbel C, Wildhagen H, Jiang X, Feußner I, Rennenberg H, Polle A (2011) The ectomycorrhizal fungus (*Paxillus involutus*) modulates leaf physiology of poplar towards improved salt tolerance. Environ Exp Bot 72:304–311. 10.1016/j.envexpbot.2011.04.008.

[ref50] Mangiafico SS (2023) Rcompanion: functions to support extension education program evaluation. version 2.4.30. Rutgers Cooperative Extension. New Brunswick, NJ. https://CRAN.R-project.org/package=rcompanion.

[ref51] Manzoni S , Trofymow JA, Jackson RB, Porporato A (2010) Stoichiometric controls on carbon, nitrogen, and phosphorus dynamics in decomposing litter. Ecol Monogr 80:89–106. 10.1890/09-0179.1.

[ref52] Mawarda PC , Le Roux X, van Elsas JD, Falcao SJ (2020) Deliberate introduction of invisible invaders: a critical appraisal of the impact of microbial inoculants on soil microbial communities. Soil Biol Biochem 148:107874. 10.1016/j.soilbio.2020.107874.

[ref53] Muller P , Li X-P, Niyogi KK (2001) Non-photochemical quenching. A response to excess light energy. Plant Physiol 125:1558–1566. 10.1104/pp.125.4.1558. 11299337 PMC1539381

[ref54] Nehls U (2008) Mastering ectomycorrhizal symbiosis: the impact of carbohydrates. J Exp Bot 59:1097–1108, 10.1093/jxb/erm334. 18272925

[ref55] Nilsen P , Borja I, Knutsen H, Brean R (1998) Nitrogen and drought effects on ectomycorrhizae of Norway spruce [*Picea abies* L. (Karst.)]. Plant Soil 198:179–184. 10.1023/A:1004399303192.

[ref56] Nosalewicz A , Okon K, Skorupka M (2022) Non-photochemical quenching under drought and fluctuating light. Int J Mol Sci 23:5182. 10.3390/ijms23095182. 35563573 PMC9105319

[ref57] Oliva J , Stenlid J, Martínez-Vilalta J (2014) The effect of fungal pathogens on the water and carbon economy of trees: implications for drought-induced mortality. New Phytol 203:1028–1035. 10.1111/nph.12857. 24824859

[ref58] Olmo M , Lopez-Iglesias B, Villar R (2014) Drought changes the structure and elemental composition of very fine roots in seedlings of ten woody tree species. Implications for a drier climate. Plant Soil 384:113–129. 10.1007/s11104-014-2178-6.

[ref59] Op De Beeck M , Troein C, Petersen C, Persson P, Tunlid A (2018) Fenton reaction facilitates organic nitrogen acquisition by an ectomycorrhizal fungus. New Phytol 218:335–343. 10.1111/nph.14971. 29297591 PMC5873446

[ref60] Parke JL , Linderman RG, Black CH (1983) The role of ectomycorrhizas in drought tolerance of Douglas-fir seedlings. New Phytol 95:83–95. 10.1111/j.1469-8137.1983.tb03471.x.

[ref61] Peay KG , Kennedy PG, Bruns TD (2011) Rethinking ectomycorrhizal succession: are root density and hyphal exploration types drivers of spatial and temporal zonation? Fungal Ecol 4:233–240. 10.1016/j.funeco.2010.09.010.

[ref62] Pollari M , Sipari N, Poque S, Himanen K, Mäkinen K (2022) Effects of Poty-Potexvirus synergism on growth, photosynthesis and metabolite status of *Nicotiana benthamiana*. Viruses 15:121. 10.3390/v15010121. 36680161 PMC9867248

[ref63] Prieto I , Roldán A, Huygens D, del Mar AM, Navarro-Cano JA, Querejeta JI (2016) Species-specific roles of ectomycorrhizal fungi in facilitating interplant transfer of hydraulically redistributed water between *Pinus halepensis* saplings and seedlings. Plant Soil 406:15–27. 10.1007/s11104-016-2860-y.

[ref64] R Core Team (2021) R: A language and environment for statistical computing. R Foundation for Statistical Computing*,* Vienna, Austria. https://www.R-project.org/.

[ref65] Read DJ (1991) Mycorrhizas in ecosystems. Experientia 47:376–391. 10.1007/BF01972080.

[ref66] Rincon A , Alvarez IF, Pera J (2001) Inoculation of containerized *Pinus pinea* L. seedlings with seven ectomycorrhizal fungi. Mycorrhiza 11:265–271. 10.1007/s005720100127. 24549345

[ref67] Sánchez F , Honrubia M, Torres P (2001) Effects of pH, water stress and temperature on in vitro cultures of ectomycorrhizal fungi from Mediterranean forests. Mycologia 22:243–258.

[ref68] Sanchez-Zabala J , Majada J, Matin-Rodrigues N, Gonzalez-Murua C, Ortega U, Alonso-Graña M, Arana O, Duñabeitia M (2013) Physiological aspects underlying the improved outplanting performance of *Pinus pinaster* Ait. seedlings associated with ectomycorrhizal inoculation. Mycorrhiza 23:627–640. 10.1007/s00572-013-0500-4. 23674120

[ref70] Sebastiana M , Tolentino Pereira V, Alcantara A, Pais MS, Bernardes SA (2013) Ectomycorrhizal inoculation with *Pisolithus tinctorius* increases the performance of *Quercus suber* L. (cork oak) nursery and field seedlings. New For 44:937–949. 10.1007/s11056-013-9386-4.

[ref69] Sebastiana M , Bernardes da Silva A, Matos AR, Alcantara A, Silvestre S, Malho R (2018) Ectomycorrhizal inoculation with *Pisolithus tinctorius* reduces stress induced by drought in cork oak. Mycorrhiza 28:247–258. 10.1007/s00572-018-0823-2. 29372408

[ref71] Shi L , Guttenberger M, Kottke I, Hampp R (2002) The effect of drought on mycorrhizas of beech (*Fagus sylvatica* L.): changes in community structure, and the content of carbohydrates and nitrogen storage bodies of the fungi. Mycorrhiza 12:303–311. 10.1007/s00572-002-0197-2. 12466918

[ref72] Shovon TA , Gagnon D, Vanderwel MC (2021) Boreal conifer seedling responses to experimental competition removal during summer drought. Ecosphere 12:e03391. 10.1002/ecs2.3391.

[ref74] Smithwick EA , Eissenstat DM, Lovett GM, Bowden RD, Rustad LE, Driscoll CT (2013) Root stress and nitrogen deposition: consequences and research priorities. New Phytol 197:712–719. 10.1111/nph.12081. 23418632

[ref75] Stenlid J (1985) Population structure of *Heterobasidion annosum* as determined by somatic incompatibility, sexual incompatibility, and isoenzyme patterns. Can J Bot 63:2268–2273. 10.1139/b85-322.

[ref76] Sterkenburg E , Bahr A, Brandström Durling M, Clemmensen KE, Lindahl BD (2015) Changes in fungal communities along a boreal forest soil fertility gradient. New Phytol 207:1145–1158. 10.1111/nph.13426. 25952659

[ref77] Sun Y , Gu JC, Zhuang HF, Wang ZQ (2010) Effects of ectomycorrhizal colonization and nitrogen fertilization on morphology of root tips in a *Larix gmelinii* plantation in northeastern China. Ecol Res 25:295–302. 10.1007/s11284-009-0654-x.

[ref78] Tedersoo L , Naadel T, Bahram M, Pritsch K, Buegger F, Leal M, Koljalg U, Poldmaa K (2012) Enzymatic activities and stable isotope patterns of ectomycorrhizal fungi in relation to phylogeny and exploration types in an afrotropical rain forest. New Phytol 195:832–843. 10.1111/j.1469-8137.2012.04217.x. 22758212

[ref79] Thorpe HC , Timmer VR (2005) Early growth and nutrient dynamics of planted *Pinus banksiana* seedlings after slash-pile burning on a boreal forest site. Can J Soil Sci 85:173–180. 10.4141/S04-011.

[ref80] Toljander JF , Eberhardt U, Toljander YK, Paul LR, Taylor AF (2006) Species composition of an ectomycorrhizal fungal community along a local nutrient gradient in a boreal forest. New Phytol 170:873–884. 10.1111/j.1469-8137.2006.01718.x. 16684245

[ref81] Unestam T (1991) Water repellency, mat formation, and leaf-stimulated growth of some ectomycorrhizal fungi. Mycorrhiza 1:13–20. 10.1007/BF00205897.

[ref82] Unestam T , Sun YP (1995) Extramatrical structures of hydrophobic and hydrophilic ectomycorrhizal fungi. Mycorrhiza 5:301–311. 10.1007/BF00207402.

[ref83] Vaario LM , Tervonen A, Haukioja K, Haukioja M, Pennanen T, Timonen S (2009) The effect of nursery substrate and fertilization on the growth and ectomycorrhizal status of containerized and outplanted seedlings of *Picea abies*. Can J For Res 39:64–75. 10.1139/X08-156.

[ref84] Van Hees PA , Rosling A, Essen S, Godbold DL, Jones DL, Finlay RD (2006) Oxalate and ferricrocin exudation by the extramatrical mycelium of an ectomycorrhizal fungus in symbiosis with *Pinus sylvestris*. New Phytol 169:367–378. 10.1111/j.1469-8137.2005.01600.x. 16411939

[ref85] Venäläinen A , Lehtonen I, Laapas M, Ruosteenoja K, Tikkanen O, Viiri H, Ilkonen V, Peltola H (2020) Climate change induces multiple risks to boreal forests and forestry in Finland: a literature review impact of drought on plants the aim of this study. Glob Change Biol 26:4178–4196. 10.1111/gcb.15183. PMC7383623 32449267

[ref86] Wu R , Cheng X, Han H (2019) The effect of forest thinning on soil microbial community structure and function. Forests 10:352. 10.3390/f10040352.

[ref87] Wubs ERJ , van der Putten WH, Bosch M, Bezemer TM (2016) Soil inoculation steers restoration of terrestrial ecosystems. Nat Plants 2:16107. 10.1038/nplants.2016.107. 27398907

[ref88] Yin D , Song R, Qi J, Deng X (2018) Ectomycorrhizal fungus enhances drought tolerance of *Pinus sylvestris* var. mongolica seedlings and improves soil condition. J For Res 29:1775–1788. 10.1007/s11676-017-0583-4.

[ref89] Zhou G , Zhou X, Nie Y et al. (2018) Drought-induced changes in root biomass largely result from altered root morphological traits: evidence from a synthesis of global field trials. Plant Cell Environ 41:2589–2599. 10.1111/pce.13356. 29879755

[ref90] Zhou T , Wang C, Zhou Z (2020) Impacts of forest thinning on soil microbial community structure and extracellular enzyme activities: a global meta-analysis. Soil Biol Biochem 149:107915. 10.1016/j.soilbio.2020.107915.

