# Supplementary material for: The effect of ectomycorrhizal fungal exposure on nursery-raised Pinus sylvestris seedlings: plant transpiration under short-term drought, root morphology and plant biomass
Source: Tree Physiol. 2024 Mar 12;44(4):tpae029. doi: 10.1093/treephys/tpae029 (PMC10990620; doi:10.1093/treephys/tpae029)
Supplement: Supporting_information_tpae029 [file supporting_information_tpae029.docx]

## Supporting Information

Article title: THE EFFECT OF ECTOMYCORRHIZAL FUNGAL EXPOSURE ON NURSERY-RAISED PINUS SYLVESTRIS SEEDLINGS: PLANT TRANSPIRATION UNDER SHORT-TERM DROUGHT, ROOT MORPHOLOGY, AND PLANT BIOMASS

Authors: GONZALO DE QUESADA, XU JIE, YANN SALMON, ANNA LINTUNEN, SYLVAIN POQUE, KRISTIINA HIMANEN, and JUSSI HEINONSALO

The following Supporting Information is available for this article:

**Table S1** Table summarizing the results of the pairwise comparison of the transpiration rate of drought-stressed and non-drought stressed treatments at the driest point and after recovery between the different seedlings exposed to EMF species, soil inoculums and control

| **Species** | **Transpiration rate at the driest point** | **Transpiration rate after the recovery period** |
| --- | --- | --- |
| *A. porphyria* | **0.02** | NS |
| *C. geophilum* | **<0.01** | **<0.01 *+*** |
| *L. laccata* | **<0.01** | NS |
| *L.* *rufus* | **0.01** | NS |
| *H. variabilis* | **<0.01** | NS |
| *P. olivaceum* | **<0.01** | **0.01** |
| *R. roseolus* | **<0.01** | NS |
| *Russula* sp. | **<0.01** | **0.02 *+*** |
| *S. bovinus* | **<0.01** | NS |
| *S. variegatus* | **<0.01** | **0.02 *+*** |
| Soil 1 | **<0.01** | NS |
| Soil 2 | **<0.01** | NS |
| Soil 3 | **<0.01** | NS |
| Control | **<0.01** | **0.03 *+*** |

Note: Significant p values (< 0.05). + indicates species with higher transpiration rate in the non-drought stress compared to the drought stress treatment

**Table S2** Table summarizing the results of the pairwise comparison of the transpiration rate and needle water potential of drought-stressed plants between the start of the drought experiment, the driest point and after recovery between seedlings exposed to EMF species, soil inoculums and the control.

| **Species** | **Needle water potential between the start and the driest point** | **Transpiration rate between the start and the driest point** | **Needle water potential between the start and after recovery** | **Transpiration rate between the start and after** **recovery** |
| --- | --- | --- | --- | --- |
| *A. porphyria* | **<0.01** | **0.02** | **<0.01** | **0.04** |
| *C. geophilum* | **<0.01** | **<0.01** | **<0.01** | **<0.01** |
| *L. laccata* | **<0.01** | **<0.01** | **0.02** | **0.02** |
| *L.* *rufus* | **<0.01** | **0.01** | **<0.01** | **<0.01** |
| *H. variabilis* | **<0.01** | **<0.01** | **<0.01** | **0.03** |
| *P. olivaceum* | **<0.01** | **<0.01** | **<0.01** | 0.06 |
| *R. roseolus* | **<0.01** | **<0.01** | 0.09 | **<0.01** |
| *Russula* sp. | **<0.01** | **<0.01** | **<0.01** | **<0.01** |
| *S. bovinus* | **<0.01** | **<0.01** | **0.01** | **<0.01** |
| *S. variegatus* | **<0.01** | **<0.01** | **<0.01** | **<0.01** |
| Soil 3 | **<0.01** | **<0.01** | 0.12 | **<0.01** |
| Soil 2 | **0.04** | **<0.01** | 0.63 | **<0.01** |
| Soil 1 | **<0.01** | **<0.01** | **<0.01** | **<0.01** |
| Control | **<0.01** | **<0.01** | **<0.01** | **<0.01** |

Note: All values where lower than at the start of the experiment. Significant p values (< 0.05) in bold (transpiration n=10; needle water potential n=3).

**Fig. S3** a) Time series displaying the average transpiration rate of seedlings exposed to EMF species compared to the control under non-drought stressed conditions. b) Time series displaying the average transpiration rate of seedlings exposed to soil inoculums compared to the control under non-drought stress conditions.

**
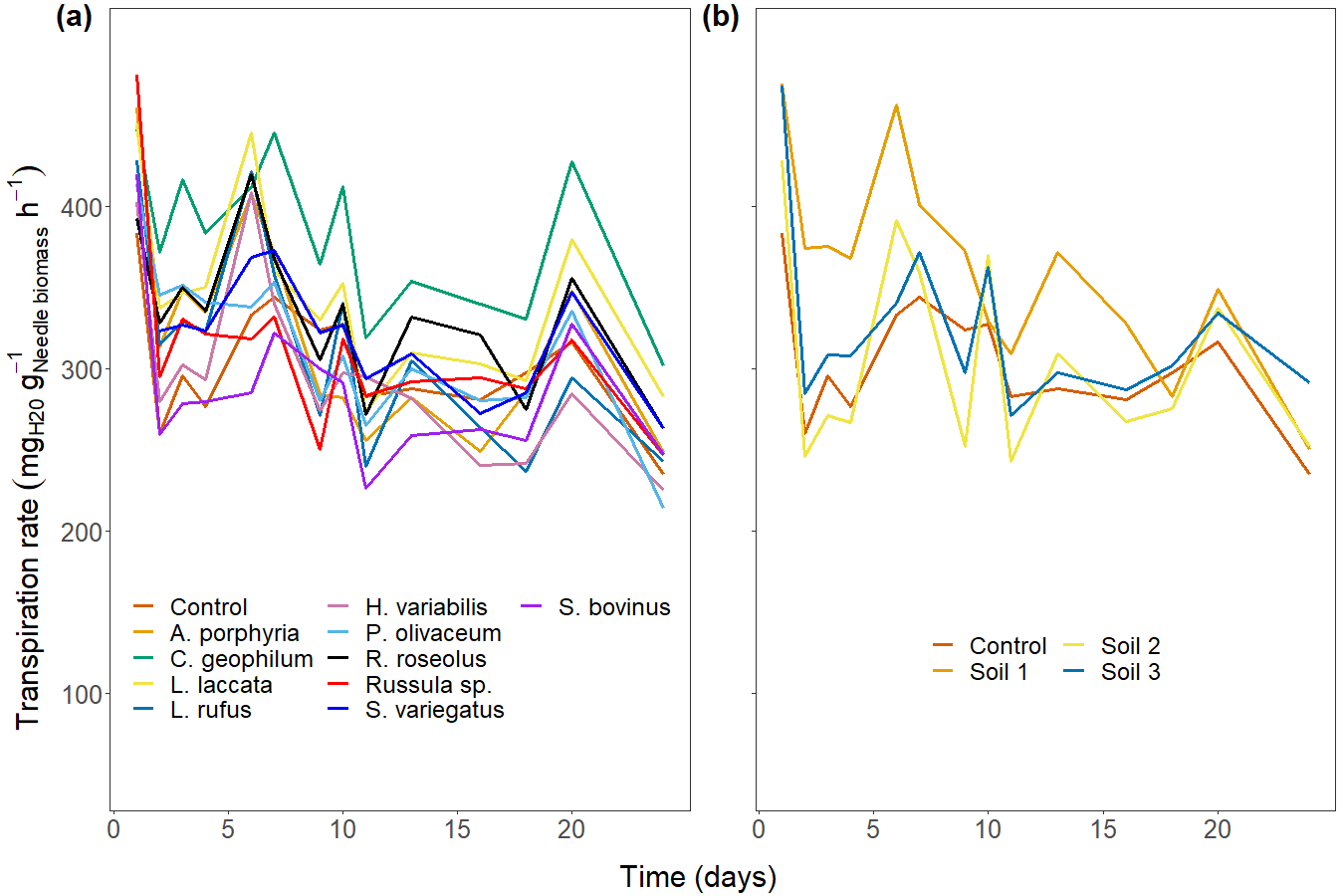
**
